# Critical Appraisal and Future Challenges of Artificial Intelligence and Anticancer Drug Development

**DOI:** 10.3390/ph17070816

**Published:** 2024-06-21

**Authors:** Emmanuel Chamorey, Jocelyn Gal, Baharia Mograbi, Gérard Milano

**Affiliations:** 1 Epidemiology and Biostatistics Department, Centre Antoine Lacassagne, University Côte d’Azur, 33 Avenue de Valombrose, 06189 Nice, France; emmanuel.chamorey@nice.unicancer.fr (E.C.); jocelyn.gal@nice.unicancer.fr (J.G.); 2 FHU OncoAge, IHU RespirERA, IRCAN, Inserm, University Côte d’Azur, CNRS 7284, U1081, 06000 Nice, France; mograbi@unice.fr; 3 Oncopharmacology Unit, Centre Antoine Lacassagne, University Côte d’Azur, 33 Avenue de Valombrose, 06189 Nice, France

**Keywords:** drug discovery, oncology, artificial intelligence, clinical trials

## Abstract

The conventional rules for anti-cancer drug development are no longer sufficient given the relatively limited number of patients available for therapeutic trials. It is thus a real challenge to better design trials in the context of new drug approval for anti-cancer treatment. Artificial intelligence (AI)-based in silico trials can incorporate far fewer but more informative patients and could be conducted faster and at a lower cost. AI can be integrated into in silico clinical trials to improve data analysis, modeling and simulation, personalized medicine approaches, trial design optimization, and virtual patient generation. Health authorities are encouraged to thoroughly review the rules for setting up clinical trials, incorporating AI and in silico methodology once they have been appropriately validated. This article also aims to highlight the limits and challenges related to AI and machine learning.

## 1. Introduction

Artificial intelligence (AI) is a general term encompassing computer programs capable of thinking like humans [1]. The main component of AI is machine learning (ML), which involves inputting data into machines and using algorithms such as Naïve Bayes, XgBoost, and Hidden Markov models [1]. Hardware has evolved to accompany the constant progress in ML. There is growing evidence to support the implementation of AI in healthcare, especially in the context of an increasingly aging population. AI has the potential to reduce the cost of current healthcare systems.

**Figure 1:**
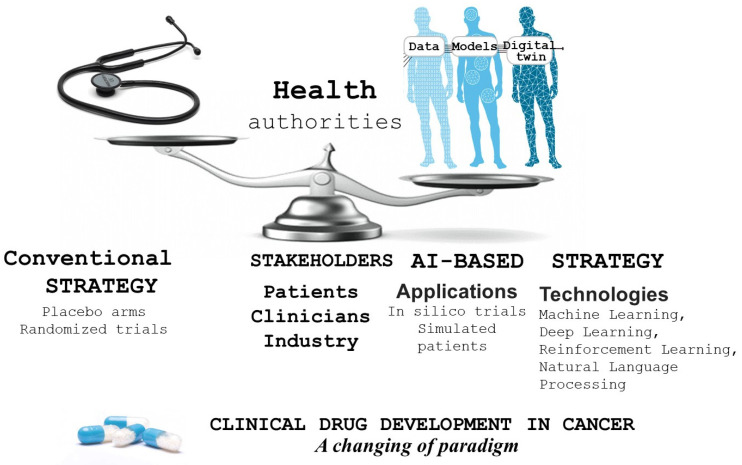
Contribution of AI in anticancer drug development.

When applied to cancer, AI can potentially radically transform clinical trial design and significantly impact preclinical drug development in several respects [2]. Its impact in this field is wide and includes the ability to more rigorously validate the hypotheses emerging from tumor profiling, define molecular mechanisms, and lead to innovative therapies. Core attributes of AI in preclinical drug development are molecular screening and target identification, thanks to the use of traditional machine learning and neural networks. More precisely, and regarding drug design, AI may generate in silico-designed molecules and analogs with specific properties [3]. We previously described the timeline for the implication of AI in anticancer drug development [4]. AI can optimize dosages, as reported by Paul et al. [5], for the use of capecitabine. These authors conducted AI-based clinical trial simulations (CTSs) to reduce toxicity while maintaining the efficacy of capecitabine. AI can also predict drug activity, as demonstrated by Claret et al. [6] in small-cell lung cancer, where a prediction of Phase III survival outcome was made using a drug–disease model framework and phase II data. CTS was also shown to be capable of designing a model representing the tumor size dynamics of gliomas under treatment with chemotherapy plus radiotherapy [7]. To date, more than 300 clinical trials have been completed or are underway that involve the application of AI to cancer treatment [8]. The main objective of this short review-position paper is not to cover the aspects related to the role of AI in drug design but rather to focus, with a critical eye, on the clinical contribution of AI in anticancer drug development (Figure 1).

## 2. Breaking the Dogma of Traditional Clinical Trial Designs

It is worth noting that the conventional rules for drug development are becoming inadequate, especially when dealing with a limited number of patients available for therapeutic trials, such as in the active and productive field of immunotherapy. In this context, there is a wide range of regimens to test, including several potentially active drug combinations where we must continue to develop biologically diverse strategies as recently reported by Perurena and coworkers for combinatorial strategies in order to target RAS-driven cancers [9]. The achievement of these objectives poses significant difficulties when applied in conventional prospective and controlled therapeutic trials, as they may miss, for instance, active drug combinations due to the limited number of patients included in the trials. Placebo-controlled studies are, for health agencies, the central pillar for validating cancer therapeutic innovations. However, these prospective therapeutic trials may face at least two major challenges. The first difficulty is the placebo itself, which can raise some ethical concerns. This could be the case when a new drug evaluated through a phase III placebo-controlled trial has already demonstrated a marked antitumor efficacy during phase II trials. This does not mean that a placebo-control add-on design should not remain applicable. The second difficulty arises when dealing with an under-represented patient population. For instance, colorectal tumors (CRC) with a microsatellite instability (MSI), may benefit from immunotherapy, as suggested by the KEYNOTE-177 trial [10]. However, under 5% of cases fall into this specific, rare group of patients, which makes it difficult to prove the benefit and implement immunotherapy in this limited population. It is thus a real challenge to improve prospective controlled therapeutic trials. This could be achieved through better designs, better selection criteria, or larger effect sizes. A promising alternative illustrated by a recent study in MSI CRC has pointed to the possibility of using databases and performing retrospective analysis with strong statistical foundations [11]. This study used a statistical tool, called the propensity score, which considers all the co-variables that could impact survival rates. This score allowed the authors to accurately compare patient subgroups who received ICIs with those who did not. This work underscores the value of AI-based propensity score methods, which encompass a variety of techniques, including machine learning algorithms such as random forests, gradient boosting machines, and neural networks [12]. These methods offer automated variable selection to mitigate the risks of overfitting and enhance the generalizability of propensity score models. On these bases, the authors reported that patients receiving third-line therapy (1813 patients) had a median overall survival that was not reached for patients on CPIs as compared to 3.5 months for those on a placebo (589 patients, HR = 0.20; 95%CI = 0.10–0.41, *p* < 0.001) and 6.5 months for those on the reference treatment (1175 patients, HR = 0.30, 95% CI = 0.15–0.60, *p* = 0.001).

The application of robust statistical tools supported by AI should thus become more and more prevalent as discussed above, with the use of propensity scores on large retrospective datasets. However, it should be kept in mind that the use of the propensity score has its proper limits. For instance, the process of propensity matching may change the population being studied. This can lead to estimates about the effects of the intervention on a different population than the initial one [13]. The central aim remains to suggest solutions to health agencies to shorten delays for patients in accessing innovative treatment. This should also significantly reduce the costs of pharmaceutical development, and also, the ethical pitfalls related to strict compliance with the prospective controlled trial strategy can be avoided.

## 3. In Silico Clinical Trials

By analogy with the expressions in vivo and *in vitro*, the term in silico was introduced to describe the numerical methods involving a mathematical approach to models that simulate biological phenomena using computers [4]. In silico trials aim to predict patient responses and outcomes using an AI-designed model. In silico clinical trials and AI technologies are distinct yet complementary concepts. AI technologies encompass a wide range of techniques (machine learning, deep learning, reinforcement learning, natural language processing) and machine learning itself includes unsupervised and supervised learnings. These computer facilities enable complex calculations to be performed quickly and automatically. In silico clinical trials involve the use of computer models, simulations, and virtual patients to predict clinical trial outcomes, as recently highlighted in the context of cancer immunotherapy [14]. These trials aim to simulate various aspects of clinical trials, such as drug effectiveness, safety, and patient responses, in a computerized environment. Altogether, AI can be integrated into in silico clinical trials to improve data analysis, modeling and simulation, personalized medicine approaches, trial design optimization, and virtual patient generation [1].

Models are generally built using data from previous trials of similar drugs, capturing individual critical parameters from a limited number of patients. Unlike conventional trial designs, in silico trials need far fewer but more informative patients and could be carried out more quickly and at a lower cost [15]. This has spawned great enthusiasm in terms of the development and clinical implementation of cancer immunotherapy. The development of kinase inhibitors for the main oncogenic drivers is also particularly active and productive [16]. However, this context may generate many similar drugs, known as “me-too” drugs, where it is difficult to distinguish the benefits for the patient versus those for drug companies. AI has the potential to markedly improve this situation and provide innovative solutions, as previously underlined by our group [4]. Clearly, in silico clinical trials can take advantage of the modeling of cumulated clinical experience and biological data generated by previously developed compounds belonging to the same category of drugs. Globally, there are two approaches to conducting in silico clinical trials for predicting outcomes of real clinical trials.

The first approach is to simulate patients by adjusting biological constants or tumor characteristics. In such a setting, Novadiscovery’s jinkō trial simulation platform [17], through a retrospective approach, successfully predicted AstraZeneca’s phase III clinical trial FLAURA2 [18]. The investigators applied a previously validated knowledge-based mechanistic model which accurately reproduced the time to progression of a clinical trial for targeted therapy of lung cancer [19]. It is interesting to note that the simulation performed with Novadiscovery’s platform took only one month to set up, while the clinical trial lasted 3 years. In particular, the simulation was able to predict that adding chemotherapy to osimertinib significantly increased progression-free survival in patients with EGFR-mutated, non-small cell lung cancer. Creemers and colleagues [14] utilized in silico cancer immunotherapy trials to construct virtual patient cohorts receiving immunotherapy, chemotherapy, or combination therapies. They successfully designed simulation models that predicted the distinct survival curves commonly associated with immunotherapies. However, it must be borne in mind that simulation remains a simulation and does not replace prospective clinical trials.

The second approach to developing in silico clinical trials is to retrospectively use big data and artificial intelligence algorithms to identify patients eligible for clinical trials and achieving this by aggregating and structuring data from patients’ medical records [20,21]. A concrete illustration of this strategy comes from the study by Eckardt and coworkers [22]. The authors demonstrated the feasibility of two different technologies of generative AI to develop synthetic clinical trial data for patients with acute myeloid leukemia and that closely mimic disease biology and clinical behaviors. The approach is particularly promising but, in reality, a specific part dedicated to real-world evidence will remain necessary in order to confront original data with AI-generated data.

Globally, in silico clinical trials hold promise for accelerating the drug development process, reducing costs, and ultimately improving patient outcomes. For instance, the use of AI provided an interesting answer to the investigators who recently questioned the real benefits of radiation therapy in gastrointestinal cancers [23].

It becomes necessary for health authorities to comprehensively revise the rules for setting up clinical trials, taking into account the potential advancements provided by AI and in silico methodology [24]. Such a strategy should maintain the notion of result reproducibility as a key criterion for acceptability by the medical and scientific community [25]. As recently stressed by Cobanaj and coworkers [26], the role of the FDA regarding AI should also be the promotion of health equity by asking investigations to transparently disclose the patient dataset composition and mandate validation on diverse patient populations.

## 4. Critical Appraisal (Table 1)

AI-designed models, primarily based on machine learning and deep-learning algorithms, offer greater flexibility in analyzing large volumes of data compared to traditional models. Although AI and machine learning approaches are promising, they are not without limitations and challenges in drug development [27,28]. For instance, the algorithms used may require large sample sizes, including labeled datasets. The development of databases across different institutions involving large network groups may be complex to set up. Interoperability standards are necessary to ensure secure and scalable data transfer. Additionally, machine learning models reach a high level of complexity and may constitute impenetrable “black boxes” with the output difficult to interpret. There is also a risk for an unavoidable widening gap between how fast technologies like AI are devised and how quickly regulatory agencies may establish legal rules for safe implementation [22].

**Table 1:** Strengths and limits of AI-based clinical trials in oncology.

**Strengths** [14]
Avoids repeated development programs. Preserves resources for true innovation. Speeds up the development of new trials.
**Limits and potential deficiencies** [27,28]
Necessitates large sample sizes with a strict control of data quality. Creates complications in terms of implementing databases across institutions (transferability), with a risk of hidden biases. May constitute impenetrable « black boxes » (interpretability). Develops virtual patients that are limited in terms of explaining complex interactions (generalizability for biology, disease, treatment).

## 5. Future Challenges

Clinical researchers, whether in industry or academia, are increasingly aware of the benefits of incorporating AI into their strategies for developing anticancer drugs. With AI, it is possible to avoid repeated development programs and thereby preserve the resources necessary for true innovation [29]. Leary and coworkers [30] have pointed out that there is a current expectation for other types of clinical research when facing the complexity of biotherapeutics and achieving this in the context of the administrative burden of regulatory requirements for new anti-cancer drug development. Interestingly, the authors proposed promoting pragmatic and affordable clinical trials. Notably, according to the authors, the tested intervention, patient selection, and follow-up could be integral parts of routine care. On the other hand, there is growing evidence that lower doses of anticancer drugs or shorter courses of precision therapy could reduce toxicity and save money [31]. To achieve this objective, the use of AI-guided clinical trials could potentially expedite the process. Due to continuous and intense changes in the clinical practice of cancer treatment, there will be the necessity to recalibrate the performance of AI-based models. As critically considered in the present position paper, AI has the potential to enable and speed up the development of new trials, through the creation of “digital” patients, which reduces the risks to human health. This does not, however, spell the end of pharmaceutical trials on humans, which remain essential and should be reformatted and not replaced by AI. In particular, all stakeholders will have to see a consistent pattern of such success before embarking on the adoption of such solutions as an alternative to prospective clinical trials. The development of AI must not constitute a source of apprehension among practitioners [28] but should lead to refocusing medical practice on guidance and support. In brief, the aim of this paper was principally to display the new possibilities offered by AI for conducting new drug development in the cancer area. However, numerous challenges and limitations do remain that we must keep in mind to provide faithful guidance that can lead to success stories.
